# Breakeven, Cost Benefit, Cost Effectiveness, and Willingness to Pay for Web-Based Versus Face-to-Face Education Delivery for Health Professionals

**DOI:** 10.2196/jmir.2040

**Published:** 2012-04-02

**Authors:** Stephen Maloney, Romi Haas, Jenny L Keating, Elizabeth Molloy, Brian Jolly, Jane Sims, Prue Morgan, Terry Haines

**Affiliations:** ^1^PhysiotherapyMonash UniversityMelbourneAustralia; ^2^Allied Health Research UnitSouthern HealthMelbourneAustralia; ^3^Health Professions Education and Educational ResearchMonash UniversityMelbourneAustralia; ^4^Health Workforce Education and Assessment ResearchMonash UniversityMelbourneAustralia; ^5^Healthy Ageing Research UnitMonash UniversityMelbourneAustralia

**Keywords:** Economics, education, training programs, teaching methods, performance

## Abstract

**Background:**

The introduction of Web-based education and open universities has seen an increase in access to professional development within the health professional education marketplace. Economic efficiencies of Web-based education and traditional face-to-face educational approaches have not been compared under randomized controlled trial conditions.

**Objective:**

To compare costs and effects of Web-based and face-to-face short courses in falls prevention education for health professionals.

**Methods:**

We designed two short courses to improve the clinical performance of health professionals in exercise prescription for falls prevention. One was developed for delivery in face-to-face mode and the other for online learning. Data were collected on learning outcomes including participation, satisfaction, knowledge acquisition, and change in practice, and combined with costs, savings, and benefits, to enable a break-even analysis from the perspective of the provider, cost-effectiveness analysis from the perspective of the health service, and cost-benefit analysis from the perspective of the participant.

**Results:**

Face-to-face and Web-based delivery modalities produced comparable outcomes for participation, satisfaction, knowledge acquisition, and change in practice. Break-even analysis identified the Web-based educational approach to be robustly superior to face-to-face education, requiring a lower number of enrollments for the program to reach its break-even point. Cost-effectiveness analyses from the perspective of the health service and cost-benefit analysis from the perspective of the participant favored face-to-face education, although the outcomes were contingent on the sensitivity analysis applied (eg, the fee structure used).

**Conclusions:**

The Web-based educational approach was clearly more efficient from the perspective of the education provider. In the presence of relatively equivocal results for comparisons from other stakeholder perspectives, it is likely that providers would prefer to deliver education via a Web-based medium.

**Trial Registration:**

Australian New Zealand Clinical Trials Registry (ACTRN): 12610000135011; http://www.anzctr.org.au/trial_view.aspx?id=335135 (Archived by WebCite at http://www.webcitation.org/668POww4L)

## Introduction

Continuing professional development for health professionals can change clinician behavior, affect patient outcomes, and influence the health of communities [1-3]. The availability of high-quality educational resources is not enough to ensure its uptake by clinicians or its delivery by educational institutions. Uptake is affected by the quality and sustainability of the product, and the accessibility and acceptability of its delivery [4].

Traditional delivery of continuing professional development has used live modalities such as lectures, tutorials, seminars, and conferences [5]. Emerging technologies enabling interactive Web-based learning environments have introduced further choice for both the provider and recipient [6]. Web-based coursework holds great promise for overcoming the key barriers of time and cost associated with the professional isolation often experienced by practitioners in rural and remote regions [7]. Evaluation of costs relative to effects would provide an important metric in assessing the value of educational resources.

In the context of the education of health professionals, there are costs associated with developing and delivering a course. Product success is determined by participant satisfaction and achievement of the defined learning targets [8]. No trials have compared the relative costs, benefits, and effectiveness of Web-based and face-to-face educational approaches for health professionals in a randomized controlled trial (RCT).

Evidence may be lacking due to the rapid expansion in the availability and acceptability of information and communication technologies used in Web-based education [9]. Furthermore, contrasting findings from economic analyses of Web-based education are often confounded by change in the assumptions that underlie the analyses, caused by changes in social expectations of access to such technologies. This creates the need to update costing models in line with any changes in social expectations—for example, some of the costs once borne by providers in providing Internet access are now paid by users who rent access to the Internet [10].

The cost effectiveness of Web-based versus face-to-face education for improving the practical skills of health professionals has not been previously reported [9-12]. This study examined the economic efficiency of Web-based versus face-to-face short-course delivery modalities in the context of falls prevention education for health professionals. The outcomes enable educational providers, health services, and health professional learners to make informed decisions about this type of investment in health professional education.

## Methods

### Design

The economic evaluation was conducted as part of an RCT comparing the educational outcomes of two short courses constructed for the education of health professionals in the skill of exercise prescription for falls prevention [13]. We conducted three analyses: (1) break-even analysis from the perspective of the education provider, (2) cost-effectiveness analysis from the perspective of the health service (the employer of the health professional), and (3) cost-benefit analysis from the perspective of the participant. We included sensitivity analyses, displaying different permutations of the variables that construct the results, to tailor the findings to different educational or workplace settings.

### Participants and Setting

Participants were eligible to enroll if they held a minimum qualification of a bachelor’s degree in any health science. We included physiotherapists, occupational therapists, nurses, and exercise physiologists working in Victoria, Australia. Participants were invited to take part in the study via the Victorian Department of Health email distribution channels.

### Measurements

#### Measurements of Cost

**Figure 1:**
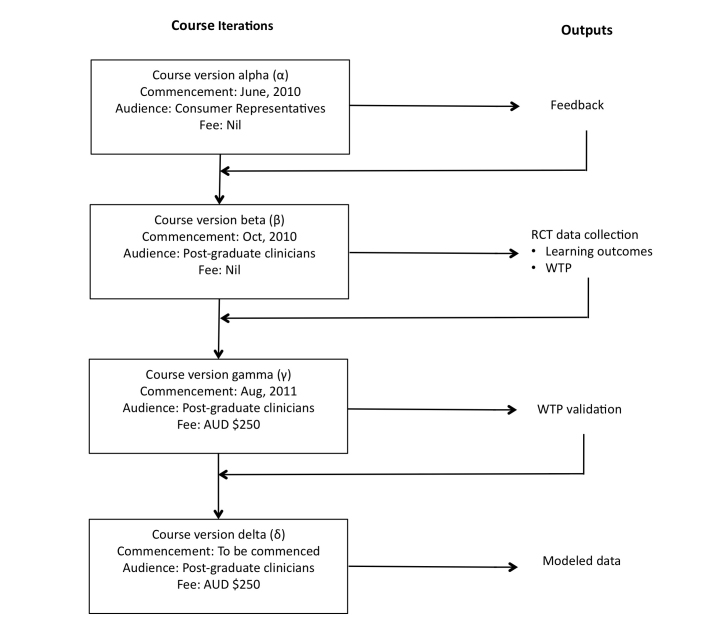
Strategy for development of the Web-based course and measurements used in the refinement and modeling of an acceptable cost framework. RCT = randomized controlled trial; WTP = willingness to pay.

The Web-based arm of the RCT was offered as an ongoing educational product by the delivering university after the initial RCT was completed. The course was developed over four iterations, shown in Figure 1. The alpha version of the program was informed by research scoping activities and delivered to representative consumers who volunteered to participate, including content specialists, educational specialists, and community members. Course version beta was delivered to practitioners who held a bachelor’s degree in a health science, forming the RCT phase and the collection of data on learning outcomes and willingness to pay. Course version gamma was a fee-paying version of the course delivered to postgraduate clinicians, allowing validation of willingness to pay data. Course version delta was based on the modeled data, most closely simulating a realistic and ongoing short-course program.

Data on the labor and capital required to provide the traditional and face-to-face education programs were collected either concurrently with the RCT or modeled afterward. Table 1 describes the approach to measuring these costs and the subsequent cost analyses [14,15]. We used market prices where known to reflect real-life costs of providing the program.

**Table 1:** Method and analysis of cost items.

Item	Delivery approach		Measurement		Determination of value	Relevant analysis		
	Face-to-face	Web-based	Model	Actual cost from RCT^a^		BEA^b^	CEA^c^	CBA^d^
Internet	No	Yes	Yes	No	Internet costs were valued by adding the course learning materials download data size (in megabytes) to the mean data size of student uploads, totaling 800 MB. We selected an existing Telstra broadband plan (accessed October 6, 2010) to cover this data cost over a 1-month period excluding set-up costs. The data limit and plan would enable the participant sufficient bandwidth and download/upload capacity to view all learning resources, complete the learning tasks, and contribute to discussion rooms over the 4-week course schedule. As some remote participants may use satellite-based Internet access, this cost was also sourced to be included in the relevant sensitivity analysis.	No	No	Yes
Transport	Yes	No	No	No	The most common mode of transportation for participants was by car. We estimated fuel costs based on the average distance participants travelled to face-to-face course venues. The average distance travelled was based on post-code data volunteered in electronic survey undertaken by RCT participants.	No	No	Yes
Opportunity cost of free time forgone	Yes	Yes	Yes	No	With wage rate providing a proxy for the opportunity cost of leisure time, we calculated a value for the participants’ time commitment by taking the mean number of hours participants required to complete the course multiplied by the hourly wage of the participant [14,15]. An hourly rate of AUD $45 an hour was used to reflect an early career physiotherapist (grade 2, year 1, Victorian Award), the largest representative group within the study demographics. In sensitivity analysis scenarios involving course participation outside of regular business hours, the wage rate was supplemented with time-and-a-half loading as would typically be experienced for clinical work outside of regular scheduled hours, ie, the weekend. These rates included 1.6 additional hours of course time for the Web-based group, as participants in this group were found within the RCT to spend significantly greater time engaged with further learning resources. Scenarios from the perspective of the health service include 17% on-costs, whereas on-costs were excluded for the CBA, as they are costs carried by the organization and not relevant from the participant’s perspective.	No	Yes	Yes
Venue rental	Yes	No	No	Yes	Venues were valued from current market prices experienced in delivering the interventions in the RCT. We set venue capacity at 20 participants for both program delivery approaches to reflect the real-life limitations of supervision and feedback time that a single tutor could provide within the practical skills practice segments of the program.	Yes	No	No
Presentation equipment	Yes	No	No	Yes	Presentation equipment included rental of a laptop computer and digital projector. Costs were valued from current market prices experienced in delivering the interventions in the RCT.	Yes	No	No

Facilitator remuneration	Yes	Yes	Yes	No	We based remuneration for the facilitator’s time in the face-to-face program delivery on the current Monash University enterprise bargaining agreement (accessed August 1, 2011) hourly sessional rate, excluding on-costs, for “repeat tutoring with a doctoral qualification.” The rate was applied to the course duration of 8 hours. Alternatives of 12 hours and 16 hours were considered in the sensitivity analysis to reflect allowances for transportation and accommodation as may be experienced with the presenter attending courses set in rural or remote locations. We based the remuneration rate for the Web-based facilitator on the tutoring Monash University sessional rate, excluding on-costs, for “repeat tutoring without doctoral qualifications.” The reduced rate for the Web-based facilitator reflected the alternative role of the Web-based facilitator, who is required to monitor and facilitate class activity, while the content is delivered by prerecorded video vignettes of a more highly qualified presenter. The Web-based facilitator was contracted for 16 hours to account for time associated with Web-based orientation enquiries from participants and accessing Web-based video submissions for feedback, which is commonly less efficient than live observation.	Yes	No	No
ICT^e^ support	Yes	Yes	Yes	No	Costs involved in the operational support of the learning platform Moodle and live presenter ICT support were not directly measured and were obtained via an internal Monash University quotation of service.	Yes	No	No
ICT licensing fees	No	Yes	No	Yes	The Web-based learning system Moodle that we used in the RCT has no current or anticipated license fees and uses open-source code.	Yes	No	No
Practical tutor assistant	Yes	No	No	Yes	Costs were valued from current market prices experienced in delivering the interventions in the RCT.	Yes	No	No
Catering	Yes	No	No	Yes	Taken from RCT face-to-face delivery costs, averaged across the three face-to-face delivery venues.	Yes	No	No
Office and stationery consumables	Yes	Yes	No	Yes	Costs were valued from current market prices experienced in delivering the interventions in the RCT.	Yes	No	No
Course support DVDs	Yes	Yes	No	Yes	Costs were valued from current market prices experienced in delivering the interventions in the RCT.	Yes	No	No
Administrative support	Yes	Yes	Yes	No	Administrative staff support was used by both delivery approaches for tasks such as processing enrollments, and mailing student materials and certificates. Costs were valued at 12 hours of Monash University Professional Staff award rate of HEW3, level 7.	Yes	No	No

^a^ Randomized controlled trial.

^b^ Break-even analysis.

^c^ Cost-effectiveness analysis.

^d^ Cost-benefit analysis.

^e^ Information and communication technology.

#### Measurements of Outcome

We derived the measure of effectiveness used in the cost-effectiveness analysis from the mark awarded for the assignment and the examination score. An assessor, blind to group allocations, marked the course assignments. Examinations comprised multiple-choice questions that were automatically graded by the software that delivered the examinations.

Mean maximum willingness to pay for the short course (beta version) was evaluated in the RCT. Participant responses were collected using an open response format and used as a proxy measure for benefit in the cost-benefit analysis [16,17]. Participants were given the option to answer a question in an anonymous Web-based questionnaire: “If this short course had not been a trial, what would be the maximum you would have been willing to pay for this course in Australian dollars (AUD)?” We asked this question in the context of four scenarios: (1) “If the course was not recognized as professional development (ongoing learning) points,” (2) “If the course had been subsidized 50% by your employer (you are indicating the presubsidized total cost),” (3) “If the course was recognized by your profession as professional development (ongoing learning) points,” (4) “If the course was recognized as prior learning (of approximately 5%–10%) toward a university postgraduate qualification, eg, master’s degree.”

### Procedure

The RCT included concealed allocation and outcome assessment by a blinded assessor for comparing face-to-face versus Web-based educational interventions.

Further details on the trial design, participants, setting, and interventions can be found in the accompanying paper of the original RCT [13].

The trial was registered with the Australian New Zealand Clinical Trials Registry, ACTRN12610000135011. We obtained ethics approval through both the Southern Health Ethics Committee and the Monash University Human Research Ethics Committee.

### Analysis

#### Break-even Analysis

The break-even analysis, from the perspective of the education provider, estimated the minimum number of participants required for the course to operate without loss to the provider. The break-even point (Q: the number of participants required) is calculated by the point where the fixed costs (FC: costs that do not vary with participant numbers) and variable costs (VC: costs that vary with the number of participants) for each mode of program delivery are equaled by the savings (S) generated by participant fees, as represented by the equation Q = FC/(S – VC).

Table 1 presents the costs considered and how they were valued. We conducted 1-way sensitivity analyses to account for possible variation in costs and savings depending on the resources and fee structure used by the education provider. Sensitivity analyses were (1) the number of hours paid for a course facilitator, (2) variation in the hourly remuneration of the facilitator, (3) class capacity (the number of students able to undertake the course at one time), (4) variation in the enrollment fee, and (5) variation in all associated costs combined.

#### Cost-Effectiveness Analysis

Health care providers often pay for their staff to attend professional development opportunities. A cost-effectiveness analysis, from the perspective of the health service, compared the relative impact of the two programs in clinical units—that is, the costs of increasing the number of trained clinicians and their measured level of clinical competence.

**Figure 2:**
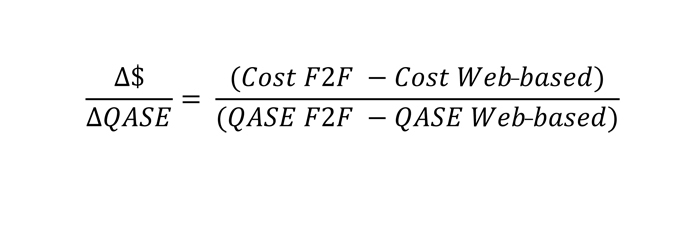
Equation for calculating cost per quality-adjusted students educated (QASE) ratio. Cost F2F = cost to the health service of the face-to-face program; Cost Web-based = cost to the health service of the Web-based program; QASE F2F = number of quality-adjusted students educated with the face-to-face program; QASE Web-based = number of quality-adjusted students educated with the Web-based program.

We calculated the cost effectiveness for each course delivery method by first determining the quality of students’ education with each method, or quality-adjusted students educated (QASE), using the formula QASE = number of students educated × the group’s average grade. In this approach, average grade is used as a surrogate for measuring the improved ability of the staff member. To account for attrition, people who did not complete the course were given a zero in this weighting calculation. This is the measurement of effect in the incremental cost-effectiveness analysis. The incremental cost per QASE was calculated using the equation in Figure 2, resulting in an incremental cost-effectiveness ratio. Table 1 lists the costs considered in the cost-effectiveness analysis.

We conducted a sensitivity analysis for the following scenarios: (1) participant attendance occurring during regular working hours, (2) participant attendance occurring during unpaid leave, (3) participant attendance occurring during participant leisure time, (4) an alternative fee structure based on profit relative to costs, and (5) whether student attrition was equivalent between the two educational approaches.

#### Cost-Benefit Analysis

A cost-benefit analysis, from the perspective of the participant, considered costs and benefits associated with the education relevant to the participant in monetary terms to enable a direct comparison.

Cost-benefit analysis requires the itemized costs experienced by each course participant along with the number of participants successfully completing the program. Net benefit, weighing the total expected costs against the total expected benefits, used the following equation: net benefit = (mean benefit face-to-face – mean cost face-to-face) – (mean benefit Web-based – mean cost Web-based), where mean benefit face-to-face is the benefit to the participant from the face-to-face method measured by the willingness-to-pay question; mean cost face-to-face is the cost to the participant to participate in the face-to-face method; mean benefit Web-based is the benefit to the participant from the Web-based method measured by the willingness-to-pay question; and mean cost Web-based is the cost to the participant in the Web-based method.

Benefit was valued in monetary terms using the participant’s willingness to pay. Sensitivity analyses included the following variables: (1) whether the participant or health service paid for enrollment, and (2) whether the course occurred including during work hours or during leisure time. To test the construct validity of the willingness to pay values obtained from RCT participants, we compared the values obtained for the course contexts with Likert ratings of overall course satisfaction using Spearman rho. In this, we hypothesized that willingness to pay values should be associated with course satisfaction ratings if the willingness to pay values reflect the construct of the benefit from participation in the program [18].

## Results

### Demographics

Participant demographics (Table 2) indicate a relatively even distribution of baseline characteristics between the face-to-face and Web-based mode participants.

**Table 2:** Chi-square test outcomes for binary data and 2-sample *t* test outcomes for years since qualification.

Demographic item		Web-based (n = 46)	Face-to-face (n = 39)	*P* value
Male gender, n (%)		10 (22%)	7 (18%)	.43
Previous falls research participation, n (%)		2 (4%)	5 (13%)	.18
Previous falls publication, n (%)		1 (2%)	0 (0%)	.35
Previous falls professional development, n (%)		11 (23%)	10 (25%)	.85
**Profession, n (%)**				
	Occupational therapy	5 (11%)	3 (8%)	.97
	Physical therapy	26 (57%)	20 (51%)	.93
	Nursing	10 (22%)	11 (28%)	.95
	Exercise physiology	4 (9%)	4 (9%)	.96
Years since qualification, mean (SD)		4.17 (1.75)	4.15 (1.56)	.66

### Randomized Trial

The RCT results indicated that there were no differences in outcomes between groups, except that Web-based education participants reported spending significantly more time (median, interquartile range of 1.0, 0.8–2.0 hours compared with 0.0, 0.0–1.0 hours) engaged with the additional learning materials than the face-to-face group (rank sum *P* = .002). The mean (SD) mark (used for calculating QASE in the cost-effectiveness analysis) for the combined examination and practical assignment grades was Web-based, 83.2% (9.9), and face-to-face, 81.6% (9.5).

### Break-Even Analysis

**Figure 3:**
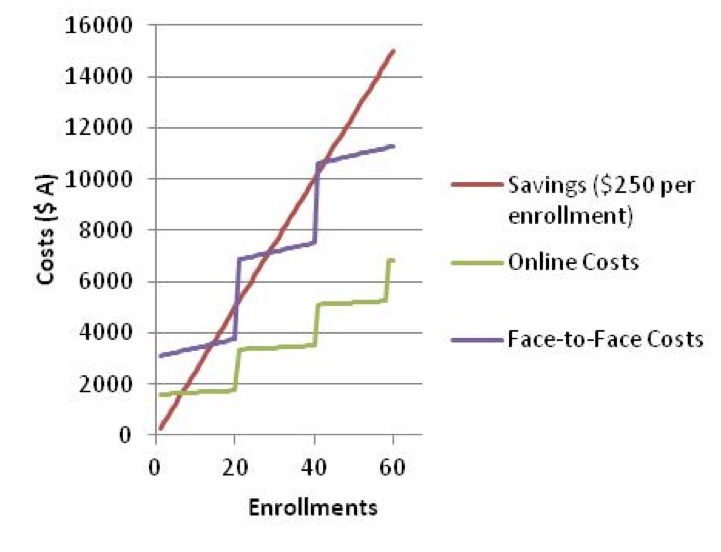
Savings versus costs for enrollment, with savings set at AUD $250 per participant and maximum class size of 20 participants.

Figure 3 presents the relationship between the costs and savings, for the primary scenario for face-to-face and Web-based delivery. Table 3 presents the fixed and variable costs considered in this analysis. Table 4 presents a sensitivity analysis, exploring the impact of variations in costs and savings. The break-even point for the primary Web-based scenario was obtained at 7 participants, whereas the primary scenario with the face-to-face delivery returned multiple break-even points (Table 5). Multiple break-even points occur in some of the sensitivity analyses when recurring fixed costs are incurred as a class reaches its enrollment capacity, causing the course to once again run at a loss until the new break-even point is reached as enrollments increase. This particular relationship, with the creation of multiple break-even points, is presented graphically for the face-to-face delivery approach in Figure 3.

**Table 3:** Fixed and variable costs (AUD $) for Web-based and face-to-face course delivery, for a maximum class size of 20.

Item	Web-based delivery		Face-to-face delivery	
	Fixed (per annum)	Variable (per participant)	Fixed (per course delivered)	Variable (per participant)
Venue			1000	
Presentation equipment rental			500	
Facilitator remuneration	840		810	
Faculty ICT^a^ support fee	500		500	
Administrative support	250		250	
Catering				25
Stationary consumables		3		5
Delivery support DVD		5		5
Total	1590	8	3060	35

^a^ Information and communication technology.

**Table 4:** Break-even and sensitivity analyses for Web-based course delivery mode.

Scenario number	Variable manipulated	Variable						Number of break-even point(s), up to 60 enrollments^a^
		Contracted facilitator hours	Presenter level ($/hour)	Maximum capacity	Other fixed costs	Variable costs	Enrollment fee (AUD $)	
1^b^		14	60	20	750	8	250	7
2	Facilitator hours	8	60	20	750	8	250	5
3	Facilitator hours	32	60	20	750	8	250	11–20, >22
4	Facilitator hours	40	60	20	750	8	250	13–20, >27
5	Facilitator hours	48	60	20	750	8	250	15–20, 30–40, 45–60
6	Presenter level	14	35	20	750	8	250	5
7	Presenter level	14	90	20	750	8	250	8
8	Presenter level	14	120	20	750	8	250	10
9	Presenter level	14	200	20	750	8	250	15–20, 30–40, 45–60
10	Class capacity	14	60	10	750	8	250	7
11	Class capacity	14	60	30	750	8	250	7
12	Class capacity	14	60	40	750	8	250	7
13	Class capacity	14	60	50	750	8	250	7
14	Class capacity	14	60	60	750	8	250	7
15	Fee	14	60	20	750	8	100	17–20, 35–40, 52–60
16	Fee	14	60	20	750	8	200	8
17	Fee	14	60	20	750	8	400	4
18	Fee	14	60	20	750	8	600	3
19	Fee	14	60	20	750	8	800	2
20	Fee	14	60	20	750	8	1000	2
21	All costs	100% increase in all associated costs (based on scenario 1)			250			14–20, >28
22	All costs	200% increase in all associated costs (based on scenario 1)			250			Doesn’t break even
23	All costs	300% increase in all associated costs (based on scenario 1)			250			Doesn’t break even
24	All costs	50% decrease in all associated costs (based on scenario 1)			250			3

^a^ Break-even points are presented as a range when multiple break-even points are relevant to the analysis. Multiple break-even points occur in some of the analyses when the new fixed costs that are incurred when a class reaches its enrollment capacity once again lift the costs above the savings. This relationship is also presented for the face-to-face program in Figure 3.

^b^ Primary analysis scenario.

**Table 5:** Break-even and sensitivity analyses for the face-to-face course delivery mode.

Scenario number	Variable manipulated	Variable						Number of break-even point, up to 60 enrollments^a^
		Contracted facilitator hours	Presenter level ($/hour)	Maximum capacity	Other fixed costs (AUD $)	Variable costs (AUD $)	Enrollment fee (AUD $)	
1^b^		9	90	20	2250	35	250	14–20, 29–40, 43–60
2	Facilitator hours	12	90	20	2250	35	250	16–20, 31–40, 47–60
3	Facilitator hours	16	90	20	2250	35	250	18–20, 35–40, 52–60
4	Presenter level	9	35	20	2250	35	250	12–20, >24
5	Presenter level	9	60	20	2250	35	250	14–20, >26
6	Presenter level	9	120	20	2250	35	250	16–20, 31–40, 47–60
7	Presenter level	9	200	20	2250	35	250	19–20, 38–40, 57–60
8	Class capacity	9	90	10	2250	35	250	Doesn’t break even
9	Class capacity	9	90	30	2250	35	250	15
10	Class capacity	9	90	40	2250	35	250	15
11	Class capacity	9	90	60	2250	35	250	15
12	Fee	9	90	20	2250	35	100	Doesn’t break even
13	Fee	9	90	20	2250	35	200	19–20, 38–40, 57–60
14	Fee	9	90	20	2250	35	400	8
15	Fee	9	90	20	2250	35	600	5
16	Fee	9	90	20	2250	35	800	4
17	Fee	9	90	20	2250	35	1000	3
18	All costs	100% increase in all associated costs (based on scenario 1			250			Doesn’t break even
19	All costs	200% increase in all associated costs (based on scenario 1)			250			Doesn’t break even
20	All costs	300% increase in all associated costs (based on scenario 1)			250			Doesn’t break even
21	All costs	50% decrease in all associated costs (based on scenario 1)			250			7

^a^ Break-even points are presented as a range when multiple break-even points are relevant to the analysis.

^b^ Primary analysis scenario.

### Cost-Effectiveness Analysis

The face-to-face educational approach, in course iteration beta, began with 68 participants, with 49 students completing the summative assessments with an average grade of 81.6%, or 38.98 QASE. Of the 67 participants who began the Web-based delivery, 44 completed the program with an average grade of 83.2%, or 35.78 QASE.

Through maintaining this attrition rate and mean grade for each delivery method, for a full class of 20 enrollments, the incremental cost-effectiveness ratio from the perspective of the health service for the primary analysis (scenario 1, Table 6) yields an incremental cost-effectiveness ratio of zero, therefore making face-to-face education more preferable from the health service perspective due to the higher rate of QASE.

**Table 6:** Sensitivity analysis of incremental cost per quality-adjusted students educated (QASE) (D$/DQASE) for Web-based (Web) and face-to-face (F2F) course delivery.

Time	Enrollment fee	Wages^a^	Backfill	Number registered	Number of completers		QASE		Costs(AUD $)		ICER^b^ per participant (AUD $)
					F2F	Web	F2F	Web	F2F	Web	
Leisure time (weekend)^c^	No	Yes	Yes	20	14	13	11.42	11.63	5000	5000	0 (F2F preferred due to higher QASE)
	Yes	Yes	Yes	20	14	13	11.42	11.63	0	0	0 (F2F preferred due to higher QASE)
Working hours	No	No	No	20	14	13	11.42	11.63	21,848	25,216	–271.62
	Yes	No	No	20	14	13	11.42	11.63	16,848	20,216	–271.62
Unpaid study leave	No	Yes	No	20	14	13	11.42	11.63	13,424	15,108	–135.81
	Yes	Yes	No	20	14	13	11.42	11.63	8424	10,108	–135.81
Conditions of scenario 1 repeated with attrition equal at 14 completers (F2F QASE = 11.42, Web-based 11.63)				20	14	14	11.42	11.63	5000	5000	0 (Online preferred due to higher QASE)
Conditions of scenario 1 repeated with alternative fee of AUD $525 applied to F2F enrollments				20	14	13	11.42	11.63	10,500	5000	443.50

^a^ Wages for the participant and backfill or replacement staff include 17% on-costs. Transport and Internet download costs are incurred by the participant. Negative dollar value indicates the value is in favor of face-to-face education.

^b^ Incremental cost-effectiveness ratio.

^c^ The primary scenario (scenario 1).

Table 6 shows a sensitivity analysis. The scenarios include manipulations of the costs carried by the health service, including the impact of the timing of course delivery. The analyses include an alternative course fee structure to account for a provider business model that calculates the fee as a percentage of profit above costs, increasing the enrollment fee for face-to-face participants. The relationship (difference in gradients) between face-to-face and Web-based costs for the primary scenarios (Figure 3) yields an inflation factor of 2.1; therefore, we adjusted the alternative face-to-face fee to AUD $525.00. The sensitivity analysis also includes a scenario of equal attrition rates between the face-to-face and Web-based approaches.

### Cost-Benefit Analysis

Table 7 presents estimated costs incurred by the participant, along with a sensitivity analysis. Net benefit to the individual participant was calculated as AUD $60.88 in favor of the face-to-face program delivery, meaning that the Web-based program would need to cost $60.88 less than the face-to-face program to create equivalent benefit for the consumer. Table 8 presents willingness to pay for each mode of program delivery. Table 9 presents sensitivity analyses of the net benefit.

Table 9 presents the values of the cost-benefit analysis. The aforementioned alternative fee structure was applied in these analyses.

**Table 7:** Primary analysis and sensitivity analyses of participant expenses (in AUD $) for Web-based versus face-to-face course delivery modes.

Participant expenses		Web-based	Face-to-face
**Primary analysis**			
	Downloads^a^	20.00	0.00
	Transport	0.00	20.00
	Fees	250.00	250.00
	Time	648.00	540.00
	Total	918.00	810.00
**Sensitivity analysis**			
	With 50% increase in fees	1043.00	810.00
	With 100% increase in fees	1168.00	935.00
	With 200% increase in fees	1418.00	1060.00
	With 50% decrease in fees	793.00	1310.00
	With 25% increase in all associated costs	1147.50	685.00
	With 50% increase in all associated costs	1377.00	1012.50
	With 25% decrease in all associated costs	688.50	1215.00
	With 50% decrease in all associated costs	459.00	607.50
	With satellite-sourced Internet	923.00	Not applicable

^a^ Download costs were calculated based on a user requiring a 1 GB data upload/download to complete the learning activities over 1 month. Costs were calculated from minimum Telstra broadband rates accessed on October 5, 2010, excluding set-up costs.

**Table 8:** Participant willingness to pay for Web-based versus face-to-face course delivery modes.

Context	Context description for willingness to pay	Web-based		Face-to-face		*P* value^b^	Correlation with overall course satisfaction: Spearman rho (*P* value)
		n^a^	mean (SD)	n^a^	Mean (SD)		
1	If course not recognized for professional development points	30	96.33 (56.37)	24	129.17 (117.25)	.41	.46 (.001)
2	If course is 50% subsidized by an employer	35	165.57 (102.16)	31	192.26 (201.46)	.46	.43 (.001)
3	If course contributes toward professional development points	36	159.72 (103.61)	30	199 (260.83)	.39	.53 (.001)
4	If coursework is recognized as prior-learning credit (5%) toward postgraduate qualification	32	190.94 (131.40)	29	314.14 (423.01)	.61	.45 (.001)

^a^ Numbers vary due to some participants not completing all fields of the survey questions.

^b^ *P* values between delivery methods obtained using single-sample mean comparison *t* test.

**Table 9:** Cost-benefit analysis results from the participant’s perspective considering varying scenarios for Web-based (Web) versus face-to-face (F2F) course delivery modes.

Scenario	Payer	Time	Enrollment fee	Opportunity cost of lost work	Benefit (willingness to pay)	Net benefit (F2F vs Web; AUD $)^a^	Alternative fee structure (F2F vs Web; AUD $)^a^
1^b^	Health service	Working hours	Yes	Yes	No	60.88	Not applicable
2		Leisure time (weekend)	Yes	No	No	168.25	Not applicable
3	Participant	Working hours	No	Yes	No	60.88	–106.37
4		Leisure time (weekend)	No	No	No	168.88	1.63

^a^ Positive values indicate more benefit in favor of face-to-face delivery mode.

^b^ The primary scenario.

## Discussion

Economic evaluation of face-to-face versus Web-based delivery has shown that the outcome depends on the stakeholder’s perspective and the conditions applied to the analysis. Web-based education is the superior approach in the break-even analysis from the perspective of the education provider. The increased costs of face-to-face delivery create increased risk of financial loss if enrollment numbers are low. This is supported by what is seen in practice, where educational institutions are reluctant to run short courses in regional or remote areas where potential enrollments are lower. This barrier supports the notion that Web-based education has the potential to be more accessible and less discriminatory [4,19]. Cost-effectiveness analysis from the perspective of the health service favors face-to-face education, but the relatively small difference in quality of the clinicians’ knowledge obtained from face-to-face and Web-based education means that the preference for an educational approach largely depends on when the course is undertaken and its impact on covering service delivery. Cost-benefit analysis, from the perspective of the participant, produced the most participant benefit from face-to-face education. Aside from the strong cost-minimization advantages to the education provider, the other analyses are contingent on the unique conditions and sensitivity analysis applied. This further highlights the novel methods used in this study, demonstrating the strong relationship between the provider, the health service, and the participant.

Economic analysis for health education has primarily focused on telemedicine technology or medical reviews by remote physicians, or has been concerned with the cost effectiveness of modalities for patient education [20,21] rather than upgrading the health professional’s skills. To our knowledge this is the first economic evaluation of competing approaches to providing continuing professional development with tested educational equivalence in an RCT including the outcomes of satisfaction, knowledge construction, and self-reported change in practice. It is possible that the findings may be somewhat context specific in terms of the content area and nature of the health disciplines involved, the specific subject of the educational materials, and the quality of the educational materials presented in the chosen medium. However, as the principles of Web-based and face-to-face education that we investigated could be transferred to other disciplines and settings, this report provides a model that can be applied to different scenarios, modified with associated unique assumptions.

This study has highlighted the precarious balance that exists between the various stakeholders involved in education programs. Our break-even analysis highlighted that using the same fee structure for both Web-based and face-to-face modalities would lead to substantially greater profits for a Web-based course assuming equal enrollments; however, this would result in lower levels of benefits to the participants in the program. However, if the university were to choose to maintain the same relative profit ratio for Web-based and face-to-face courses, they would be able to offer the Web-based course at a lower cost to gain equivalent educational outcomes and participant benefit; society would therefore gain a greater volume of skilled professionals for a lower cost.

Potential miscellaneous benefits from each delivery method were excluded from the analysis due to the difficulty in quantifying them. For example, it is hypothesized that face-to-face education may foster stronger feelings of an education community, socialization, and networking opportunities [22], whereas Web-based education may provide better maintenance of corporate knowledge that is often lost with key personnel in short-course training and lost to the profession in situations such as retirement or maternity leave [23].

Other limitations that may have affected the findings of the analysis relate to the original RCT, the modeling of data, and underlying assumptions in the analysis formulas. The RCT measured the learning outcomes and change in practice behavior approximately 1 week following the conclusion of the training program. We do not know whether the effect of the training would have been different if the effects had been measured over a longer time frame. We also do not know whether the change in clinician practice behavior will result in real change in patient outcomes over time. We modeled data for the analysis on the fourth iteration of the training program, excluding costs involved in constructing the programs and additional expenses from “teething errors” in delivering the course with unfamiliar staff. These issues are particularly relevant to the introduction of the Web-based modality. Phelps et al [11] recognized that setting up a Web-based course, or converting an existing course to a Web-based modality, requires a large human capital investment. Our experience in creating the course resources for the RCT reflected this sentiment, but we also found that this argument was true of beginning a face-to-face program. Audiovisual resources for the Web-based course were ideally created for both course delivery methods, but rather than being used as direct student resources in the face-to-face delivery, they were used as a way of training face-to-face presenters and maintaining corporate knowledge. We anticipate this to be cost prohibitive for many organizations without significant information and communication technology (ICT) infrastructure or with staff unfamiliar with using basic computers and software to commence a Web-based course [9,10], particularly with the expectation of making short-term savings.

Each formula used in the analysis involves calculations based on assumptions. These assumptions may be invalid depending on the context of the analysis or variation in reader perspective. Wells [24], Phelps et al [11], and Rumble [12] all reported that the shifting assumptions in information communication technologies affect the ability to contrast studies investigating the use of these technologies in education, such as who pays for what costs, the existing ICT infrastructure, and the degree of assumed user ICT literacy. For example, Rumble’s [12] investigation required the university to supply computers to all students involved in the study. It could be argued that students are now assumed to have access to their own personal computer or readily accessible university or workplace computer facilities. In our study we required students to have their own access to a computer and the Internet.

The issue of who pays for what costs will naturally affect the economic evaluations. Our cost-benefit analysis assumed that students who did not complete the program did not obtain benefit from the small portion of the program that they may have completed. We averaged transport costs among the study cohort and limited them to transport by car. In reality, participants would have experienced a significant range of transport costs, which naturally depend on the location of the face-to-face delivery venue. The formulas used to calculate cost benefit and cost effectiveness require data on participant attrition in each delivery method. Participants were enrolled in the training program of the RCT at no cost. This lack of financial commitment to the program is likely to have negatively affected student attrition. This factor may have applied more to the Web-based education intervention, as it required a prolonged time commitment from students in its delivery over a 4-week period.

Although we do not know the true generalizability of the findings, the transparency of this analysis will assist decision makers to tailor the findings to their area of interest and delivery setting, whether it is undergraduate or postgraduate education. Key factors in this decision making would include the ICT capabilities of the provider, similarity of their desired course to the constructivist pedagogy used in the practical skill-teaching methods of this analysis, and other particular circumstances or resources of the provider.

We used willingness to pay as a proxy for determining participants’ perceived benefit of participating in the program. Willingness to pay may have been underreported if participants considered that their responses might potentially influence the cost of education in a market in which they are the consumer. We expect that this bias would have minimal impact on the analysis between the two interventions, as the effect is likely to be equal between the two groups. Another potential bias influencing the willingness to pay values, and therefore the benefit and cost effectiveness of the delivery methods, is a possible perception that Web-based education produces greater savings for the educational institution, and that these savings should be passed on to the participant via savings in course fees [25]. Drummond et al [18] noted that it is often impossible to validate willingness to pay values within economic analyses due to the modeling of data being commonly based on theoretical or hypothetical products or services. This study was able to provide values based on programs purposefully designed for equivalent content delivery and experienced by the participants who provided the willingness to pay values. Construct validity of the willingness to pay values obtained was demonstrated through the moderate degree of correlation between the willingness to pay values and the participants’ ratings of overall satisfaction (Table 9). Furthermore, the fee (AUD $250) used as the primary scenario in the economic evaluations was used in the fee-paying gamma version course iterations, a course that reached its full enrollment capacity (20 participants), indicating that this fee was a viable real-life fee structure and is comparable with fees for other Web-based professional development programs of equivalent length on the market.

QASE as represented in the cost-effectiveness analysis assumes that the health care service costs are the same for each delivery group. This may be debated depending on whether the participant requires study leave to complete the course or completes the course out of work hours. QASE and cost per QASE (Δ$/ΔQASE) is also grounded in the assumption that a clinician with better skills in falls risk client management will be a greater asset to the health service, producing better health outcomes for the clients.

Education providers vary in their opinion as to what practical class enrollment capacity is manageable in a face-to-face delivery environment. An increase in class capacity will often result in an overall decrease in fixed costs, affecting the result of a break-even analysis [26]. This is also true of Web-based education, although given the asynchronous nature of the student and teacher interaction, Web-based education is arguably less restricted by the course duration in providing supervision and feedback on participant practice, and in turn in producing greater flexibility in discerning the maximum class capacity.

We have highlighted several areas for further research in exploring the economics of delivery approaches to continuing professional development. These include further investigation into the attrition rates when contrasting free versus full fee paying. Investigating the allocation of costs in continuing professional development for health care professionals, particularly the contribution to the course fee between the participant and the health service, would allow greater insights into the underlying assumptions in analyses. More broadly, we would encourage research into using this approach (ie, break-even analysis from the perspective of the provider, cost-effectiveness analysis from the perspective of the health service, and cost-benefit analysis from the perspective of the participant) to explore other educational approaches. Ultimately, an RCT would be conducted to investigate what may be the largest assumption in this study, that a more highly skilled clinician can affect health outcomes. For example, does the educational program to increase clinicians’ management of falls-risk clientele, studied in this evaluation, have the ability to reduce falls in the community?

### Conclusions

Economic analysis of Web-based versus face-to-face training for improving the clinical performance of health professionals varies depending on the type of analysis and stakeholder perspective undertaken. The Web-based educational approach was clearly more efficient from the perspective of the education provider. In the presence of equivocal results for comparisons from other stakeholder perspectives, it is likely that providers will seek to deliver education via a Web-based medium in preference to an alternative face-to-face approach.

With both Web-based and face-to-face delivery modalities offering their own unique strengths and weaknesses, it is important for decision makers to consider the application of these findings to their goals, their risk management capabilities, and their role in the delivery of high-quality health care to the community through the effective and efficient education of health service clinicians.

## Abbreviations

ICT: information and communication technology
QASE: quality-adjusted students educated
RCT: randomized controlled trial
